# A statistical framework for QTL hotspot detection

**DOI:** 10.1093/g3journal/jkab056

**Published:** 2021-02-26

**Authors:** Po-Ya Wu, Man-Hsia Yang, Chen-Hung Kao

**Affiliations:** 1 Institute of Statistical Science, Academia Sinica, Taipei 11529, Taiwan, Republic of China; 2 Crop Science Division, Taiwan Agricultural Research Institute, Council of Agriculture, Taichung 41362, Taiwan, Republic of China; 3 Department of Agronomy, National Taiwan University, Taipei 10617, Taiwan, Republic of China

**Keywords:** QTL hotspots, pleiotropic traits, permutation tests, genetical genomics, genetic correlation, LOD thresholds

## Abstract

Quantitative trait loci (QTL) hotspots (genomic locations enriched in QTL) are a common and notable feature when collecting many QTL for various traits in many areas of biological studies. The QTL hotspots are important and attractive since they are highly informative and may harbor genes for the quantitative traits. So far, the current statistical methods for QTL hotspot detection use either the individual-level data from the genetical genomics experiments or the summarized data from public QTL databases to proceed with the detection analysis. These methods may suffer from the problems of ignoring the correlation structure among traits, neglecting the magnitude of LOD scores for the QTL, or paying a very high computational cost, which often lead to the detection of excessive spurious hotspots, failure to discover biologically interesting hotspots composed of a small-to-moderate number of QTL with strong LOD scores, and computational intractability, respectively, during the detection process. In this article, we describe a statistical framework that can handle both types of data as well as address all the problems at a time for QTL hotspot detection. Our statistical framework directly operates on the QTL matrix and hence has a very cheap computational cost and is deployed to take advantage of the QTL mapping results for assisting the detection analysis. Two special devices, trait grouping and top $\gamma_{n,\alpha }$ profile, are introduced into the framework. The trait grouping attempts to group the traits controlled by closely linked or pleiotropic QTL together into the same trait groups and randomly allocates these QTL together across the genomic positions separately by trait group to account for the correlation structure among traits, so as to have the ability to obtain much stricter thresholds and dismiss spurious hotspots. The top $\gamma_{n,\alpha }$ profile is designed to outline the LOD-score pattern of QTL in a hotspot across the different hotspot architectures, so that it can serve to identify and characterize the types of QTL hotspots with varying sizes and LOD-score distributions. Real examples, numerical analysis, and simulation study are performed to validate our statistical framework, investigate the detection properties, and also compare with the current methods in QTL hotspot detection. The results demonstrate that the proposed statistical framework can effectively accommodate the correlation structure among traits, identify the types of hotspots, and still keep the notable features of easy implementation and fast computation for practical QTL hotspot detection.

## Introduction

The quantitative trait loci (QTL) mapping experiments have been performed for traditional traits (such as yield and quality in rice, weight and body fat percentage in animals, and diabetes and hypertensions in human) and molecular traits (such as gene expression or protein levels using the newly developed microarray technique) to explore the genetic mechanisms of these traits in various organisms and many areas of biological studies. When performing on traditional traits, a single experiment can produce abundant marker genotypes but usually consider only a few traits, say about 10–20 traits, in the population, since measuring traditional traits can be time-consuming and a costly process. On the contrary, when the experiment is conducted on molecular traits (called the genetical genomics experiment), with the aid of a high-throughput molecular biology techniques, it can not only produce abundant marker genotypes but also generate thousands of molecular traits for the individuals at a time (Jansen and Nap 2001; Brem *et al.* 2002). To detect the QTL for these traits, many statistical methods have been proposed to analyze the QTL mapping data for the estimation of QTL parameters, including the QTL effects and positions, epistasis among QTL, heritabilities, etc. (Lander and Botstein 1989; Haley and Knott 1992; Jansen 1993; Zeng 1994; Kao *et al.* 1999; Sen and Churchill 2001; Xu 2003; Broman *et al.* 2003; Kao 2006; Lee *et al.* 2014; Wei and Xu 2016; da Silva Pereira *et al.* 2020). In QTL mapping for either the traditional or molecular traits, it has been observed that QTL are highly abundant in some of the genomic regions and that QTL responsible for correlated traits are frequently clustered closely together in some specific genetic regions as compared to other regions (Goffinet and Gerber 2000; Schadt *et al.* 2003; Chardon *et al.* 2004; West *et al.* 2007; Breitling *et al.* 2008; Wu *et al.* 2008; Wang *et al.* 2014; Basnet *et al.* 2015; Yang *et al.* 2019). These regions enriched in QTL are referred to as QTL hotspots, and, statistically, they harbor a significantly higher number of QTL than expected by random chance. It has been noted that the phenomenon of QTL hotspots may have several causes, such as: QTL with high allelic polymorphisms have a greater chance of being detected in different crosses and environments; pleiotropic or closely linked QTL that control correlated traits are frequently co-localized in the same regions in different experiments (Falconer and Mackay 1996; Zhao *et al.* 2011; Vuong *et al.* 2015; Mengistu *et al.* 2016; Zhang *et al.* 2020). As the QTL hotspots can lead to identifying genes that affect the traits of interest, and further help to build networks among QTL hotspots, genes, and traits, the QTL hotspot detection analysis at genome-wide level has been a key step toward deciphering the genetic architectures of quantitative traits in genes, genomes, and genetics studies (Breitling *et al.* 2008; Fu *et al.* 2009; Neto *et al.* 2012; Wang *et al.* 2014; Yang *et al.* 2019).

Genome-wide QTL hotspot detection first needs to collect data with many QTL to proceed with the detection analysis. So far, both the genetical genomics experiments and public QTL databases can provide the data sets with many QTL for the hotspot analysis, but note that these two data sources have different structures. The genetical genomics experiment contains individual-level data (containing the original marker genotypes and many molecular traits) that allow to detect thousands of QTL in a single experiment. And public database (such as GRAMENE, Q-TARO, Rice TOGO browser, PeanutBase, and MaizeGDB) curates thousands of summarized QTL data (containing the detected QTL, trait names, and reference sources without any individual-level data) for various traditional traits from numerous independent QTL experiments. Using these two types of data, several statistical methods mainly based on permutation tests have been proposed to detect QTL hotspots. West *et al.* (2007), Wu *et al.* (2008), Li *et al.* (2010), Breitling *et al.* (2008), and Neto *et al.* (2012) developed statistical methods to detect QTL hotspots using the genetical genomics experiments. The methods of West *et al.*, Wu *et al.*, and Li *et al.* (the *Q*-method) first perform QTL mapping at all genomic positions for all traits to construct a QTL matrix. Then, they permuted the row elements of the QTL matrix separately by trait to compute the thresholds of hotspot size (in terms of the number of QTL) for assessing the significance of QTL hotspots. As these methods do not account for the correlation structure among traits, the thresholds are severely underestimated, leading to the detection of too many spurious hotspots (Breitling *et al.* 2008; Neto *et al.* 2012). To consider the correlation structure among traits, Breitling *et al.* (2008) permuted the individual-level data by shuffling the all traits relative to genotypes to generate numerous permuted data sets and then performed QTL mapping on each of the permuted data sets to obtain the QTL matrices and determine the hotspot thresholds in hotspot detection. The way of permutation in Breitling *et al.* (the *N*-method) can preserve the correlation structure among traits and provide stricter thresholds to prevent spurious hotspots due to non-genetic correlation. However, without considering the magnitude of the LOD scores for QTL, biologically interesting hotspots composed of a moderate-to-small number of QTL with strong LOD scores may be missed and discarded as nonsignificant (Neto *et al.* 2012). To further consider the LOD scores of QTL in hotspot detection, Neto *et al.* (2012) adopted the same permutation schemes and took the magnitude of LOD scores into account to compute a series of LOD thresholds for different hotspot sizes. The approach of Neto *et al.* (the *NL*-method) can effectively discover hotspots containing a moderate-to-small number of QTL with strong LOD scores. Later, using the summarized QTL data in public databases, Yang *et al.* (2019) proposed a statistical procedure that operates on the QTL matrix with trait grouping to tackle the issue in genome-wide QTL hotspot detection. As well noted, the methods by permuting the individual-level data (the *N*- and *NL*-methods) involve repeated the QTL mapping analysis in each permutation and will suffer from the problem of computational intractability and may require parallel computations to complete the analyses (Neto *et al.* 2012). On the contrary, the methods by permuting the QTL [or expected QTL frequency (EQF)] matrix (the *Q* and Yang *et al.* methods) need to perform QTL mapping analysis only once in the whole procedure and, therefore, can offer the advantage of easy implementation and very cheap computation for QTL hotspot detection (Yang *et al.* 2019).

In this article, we introduce a general statistical framework that can handle both types of data as well as take care of all the above concerns, including the correlation structure among traits, the magnitude of LOD scores of QTL in a hotspot and computational cost, for QTL hotspot detection. Our statistical framework operates on the QTL matrix or the EQF matrix and hence is very cheap in computation. By taking the advantages of using the individual-level data in genetical genomics experiment, the estimates of QTL parameters and the LOD scores at every position for all traits can be obtained by the QTL mapping technique and used to benefit the QTL hotspot analysis. Our statistical framework attempts to take the QTL mapping results into account to address the concerns and facilitate the hotspot detection. Two special devices, trait grouping and top $\gamma_{n,\alpha }$ profile, are deployed in the framework. In trait grouping, we first show that traits controlled by the tightly linked or pleiotropic QTL (tightly linked or pleiotropic traits) may have arbitrary values at their phenotypic or genetic correlations, and hence we use the estimated QTL positions, rather than the phenotypic or genetic correlations among traits, to make inference about the tightly linked and/or pleiotropic traits for trait grouping. Then, the permutation algorithm of Yang *et al.* (2019) is deployed to randomly shift the tightly linked and/or pleiotropic QTL together along the genome separately by trait group, accounting for the correlation structure among traits, to compute a series of EQF thresholds, $\gamma_{n,\alpha }s$, for hotspot detection. For each hotspot, we profile the top $\gamma_{n,\alpha }$ thresholds and use the profile to outline the LOD-score pattern across the different LOD thresholds. The top $\gamma_{n,\alpha }$ profile can then serve to characterize the types of hotspots with varying sizes and LOD-score distributions, so as to have the ability to assess the small and moderate hotspots with strong LOD scores. In this way, our framework can overcome the underestimation of threshold arising from ignoring the correlation structure among traits and also identify the different types of hotspots with very low computational cost during the detection process. Numerical analysis, simulation study, and real examples are conducted to explore the patterns of genetic correlations between closely linked and/or pleiotropic traits, investigate the properties of the proposed statistical framework, and assess the performances and compared with the current approaches. We demonstrate that the proposed statistical framework can deal with both types of data, effectively accommodate the correlation structure among traits, hotspot sizes, LOD score distributions among QTL, and still keep the notable features of easy implementation and fast computation to benefit hotspot detection.

## Materials and methods

Our statistical framework aims to operate on both the summarized data from public databases and individual-level data from genetical genomics experiments for QTL hotspot detection. The key and basic idea of our framework is to perform permutation analysis on the QTL matrices or EQF matrices, rather than on the original individual-level data, and to well utilize the QTL mapping results in the process of QTL hotspot detection. Since the summarized data are an intermediate component in the detection process of using the original individual-level data, the framework for operating on the individual-level data to detect the QTL hotspots is first described, and that for summarized data will follow without additional treatment. In the following, how the QTL matrices are constructed from the different LOD thresholds in QTL mapping using the individual-level data is first described. Then, we convert the QTL matrices into the EQF matrices by assuming that the QTL position is normally distributed over its own QTL interval. After that, we show that using the estimated QTL positions is more effective than using the phenotypic or genetic correlations in the inference of closely linked and/or pleiotropic traits, which will be grouped together into the same trait groups for the permutation analysis. Then, the permutation algorithm of Yang *et al.* (2019) that permutes the closely linked and/or pleiotropic QTL together across the genome in each trait group is outlined and applied to the QTL matrices or EQF matrices, and each computes a series of EQF thresholds, $\gamma_{n,\alpha }s$, varying from strict to liberal, for assessing the hotspot significance. For every hotspot, we profile the top $\gamma_{n,\alpha }$’s thresholds across the different EQF matrices and use the top $\gamma_{n,\alpha }$ profile to identify the different types of QTL hotspots with varying sizes and LOD-score distributions. Numerical analysis, simulation study, and real examples are followed to validate our statistical framework, investigate the detection properties, and also compare with the current methods in QTL hotspot detection.

### LOD scores and QTL matrices

A variety of QTL mapping methods have been proposed to estimate the genetic architecture parameters of quantitative traits (see *Introduction*). Using these methods, for each trait, a (partial) likelihood-ratio test (LRT) statistic or an LOD score (1 LOD ≈ 4.6 LRT) is calculated to test for the existence of a QTL at every genomic position. The LOD scores at every position for all traits can be recorded into an LOD score matrix. Then, given a predetermined LOD threshold for the test, the LOD score matrix can be converted into a QTL matrix by assigning 1 to the detected QTL positions and 0 otherwise. Several analytical, computational, and empirical approaches have been developed to determine the appropriate LOD threshold (Lander and Botstein 1989; Churchill and Doerge 1994; Piepho 2001; Chang *et al.* 2009; Guo 2011; Kao and Ho 2012). In general, obtaining thresholds using the analytical approaches, such as the Gaussian processes by Chang *et al.* (2009), Guo (2011), and Kao and Ho (2012), has a very cheap computational cost and may require the assumption of normal distribution, and using the permutation test is robust to departures from normal assumption but needs to handle the problem of computational intractability. In the context of hotspot detection analysis, for ease of computation, it is possible that the LOD thresholds can be determined based on empirical experience or by using the Gaussian process without bothering to use the permutation tests. Given an LOD threshold, the estimated QTL positions, their confidence intervals (constructed by using the asymptotic SD), and LOD support intervals (Lander and Botstein 1989) can be obtained. Note that, in the public databases, neither LOD scores nor the confidence intervals of QTL positions are provided, and only the flanking markers of the detected QTL are recorded. Here, we called the confidence interval of a QTL position or the marker interval containing a QTL interval. Then, we can use a QTL matrix (an atypical matrix) with column dimension equivalent to the genome size and row dimension corresponding to the number of traits to summarize the QTL intervals for all traits as follows: for each trait, we mount the QTL intervals onto the elements of a row array as follows: each QTL interval stands for an element of the length as its width at the corresponding position, and a value of one is given to the element. The remaining elements will be treated as zeros. Combining the arrays for all traits will form a QTL matrix, whose elements are either one or zero with unequal lengths (see Figure 1 in Yang *et al.* 2019 for graphical illustration). In this way, for a range of LOD thresholds from relaxed to conservative, say LOD thresholds of 3–8 (by one increment), we can construct several (six) corresponding QTL matrices for operation. The natural choices for the relaxed and conservative LOD thresholds are the single-trait QTL mapping threshold controlling genome-wide error rate (GWER) for one trait and a multiple single-trait QTL mapping threshold controlling GWER across all traits, respectively, as suggested by Neto *et al.* (2012). The QTL matrices constructed with higher LOD thresholds will contain fewer QTL but of larger LOD scores and produce smaller hotspot size thresholds. Such a property can be applied to consider both hotspot size and LOD-score distribution of QTL for a hotspot in the detection analysis.

**Figure 1:**
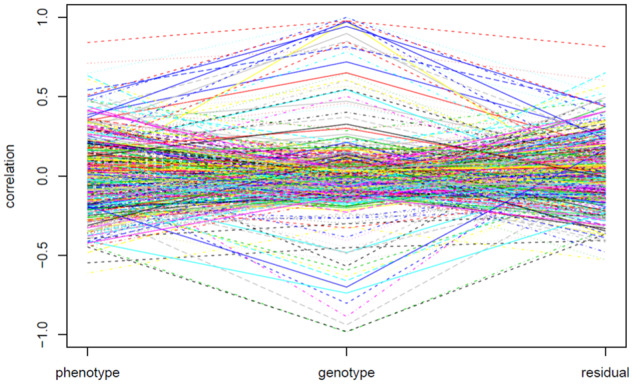
Phenotypic, genetic, and residual correlations of the 500 randomly selected pairwise traits that controlled by the QTL with LOD scores larger than 3 in the yeast data set.

### Expected QTL frequency, EQF matrices, and EQF architecture

**Figure 2:**
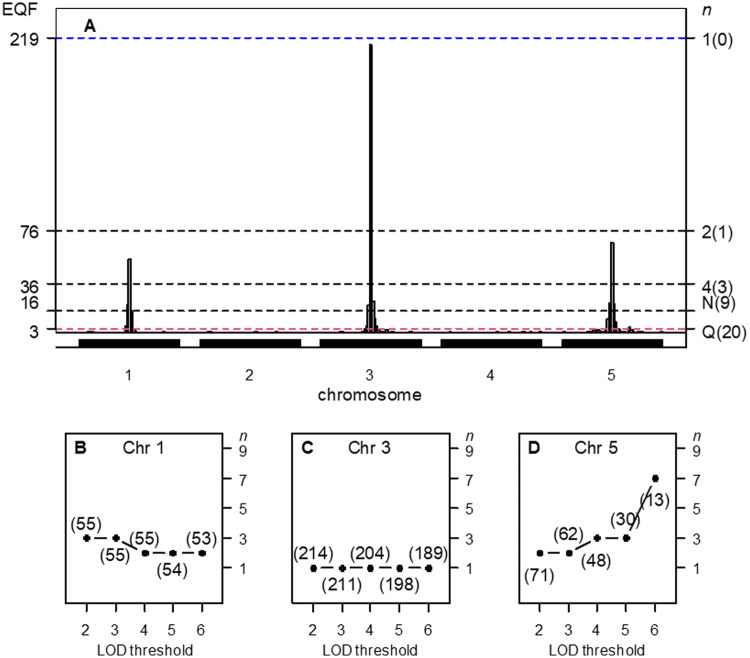
(A–D) The hotspot architecture and the top $\gamma_{n,\alpha }$ profiles of the three simulated hotspots across the 2- to 6-LOD EQF architectures. (A) Inferred hotspot architecture using a single-trait permutation LOD threshold of 2.47 corresponding to a GWER of 5% of falsely detecting at least one QTL somewhere in the genome. The hotspots on chromosomes 1, 3, and 5 have sizes 55, 214, and 67, respectively. The thresholds $\gamma_{n,0.05}$ are coordinately represented by the left and right axes. The left axis denotes the values of EQF, and the right axis denotes the values of *n*. The dashed line at count 16 corresponds to the hotspot size threshold at a GWER of 5% according to the *N*-method. The dashed line at count 3 corresponds to the *Q-*method’s 5% significance threshold. The thresholds $\gamma_{1,0.05}$, $\gamma_{2,0.05}$, and $\gamma_{4,0.05}$ obtained by the proposed procedure are 219, 76, and 36, respectively. The number in the bracket on the right axis denotes the number of detected hotspots. (B) The top $\gamma_{n,\alpha }$ profile for hotspot A shows a decreasing pattern with the value of *n* decreasing from 3 to 2 over the 2- to 6-LOD EQF architectures, indicating that hotspot A contains relatively more QTL with large LOD scores. (53): the hotspot size (number of QTL) is 53 and the top $\gamma_{n,0.05}$ threshold is $\gamma_{2,0.05}$ in the 6-LOD EQF threshold. (C) The top $\gamma_{n,\alpha }$ profile for hotspot B shows a flat pattern with *n *=* *1 for all the EQF architectures, indicating that hotspot B containing QTL with balanced LOD scores. (D) The top $\gamma_{n,\alpha }$ profile for hotspot C shows an increasing pattern with the value of *n* increasing from 2 to 7 over the five EQF architectures, indicating that hotspot C contains relatively fewer QTL with large LOD scores. Results are based on 1,000 permutations. Q: The *Q-*method; N: The *N*-method.

**Figure 3:**
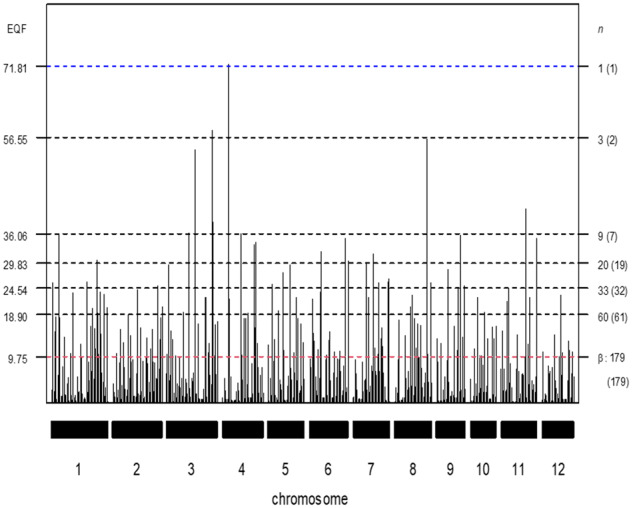
The EQF architectures along the 12 chromosomes and the hotspots detected under different EQF thresholds ($\gamma_{n,0.05}$) associated with their qFreq(*n*) statistics at GWER of 5% the GRAMENE rice dataset. The thresholds $\gamma_{n,0.05}$ are coordinately represented by the left and right axes. The left axis denotes the values of EQF, and the right axis denotes the values of *n*. The blue line corresponds to the EQF threshold $\gamma_{1,0.05}=71.81$ for detecting at least one hotspot, and there is one (the number in the bracket) hotspot detected with $\gamma_{1,0.05}$. Similarly, $\gamma_{3,0.05}=56.55$ for detecting at least three hotspot, and there are two hotspots detected with $\gamma_{3,0.05}$. The red line shows $\gamma_{179,0.05}=9.75$ for detecting at least 179 hotspots, which approximately corresponds to β (the EQF threshold of the *Q*-method), and there are 179 significant hotspots with $\gamma_{179,0.05}$.

We assume that *m* QTL matrices have been constructed from the LOD score matrix using the *m* different LOD thresholds ($L_{1},\;L_{2},\ldots ,L_{m}$). We now take one QTL matrix as an example to show how to compute the EQF of a bin and to construct the EQF matrix. The remaining EQF matrices of the other QTL matrices can be obtained in the same way. Consider that there are T traits each mapped for $N_{1},\;N_{2},\ldots ,\;N_{T}$ QTL (intervals), respectively, where *N*$=N_{1}+N_{2}+\cdots +N_{T}$ is the total number of QTL. Assume that the genome is divided into *W* sequential equally spaced bins, each with the same size △ (say △=1 or 2 cM), for QTL hotspot analysis. For a bin $\left(x,x+△\right)$ and a QTL interval $\left(a,b\right)$, where x, a, and b denote the genomic positions, they may have an overlap or no overlap. When there is an overlap, there is a probability that the QTL is localized in the bin, and the QTL will contribute a probability to the EQF value of this bin. Such a QTL will be referred to as a contributive QTL of a bin. We further assume that the QTL position is normally distributed over its own QTL interval to compute the contributed probability (EQF value) in a bin. Now let $f_{tw}$ denote the EQF value of the *w*th bin between x and x+△ for the *t*th trait, where t=1, 2,…,T and w=1, 2,…,W. We have

(1) $$f_{tw}=\sum_{i=1}^{N_{t}}\{\int_{u_{i}}^{v_{i}}N_{x}(p_{i},s_{i}^{2})\;d\left(x\right)\},$$

where $N_{t}$ is the number of contributive QTL of the *t*th trait, $\left(u_{i},v_{i}\right)$ is the overlap region, $p_{i}$ is the estimated QTL position (LOD peak) of the contributed QTL, and $s_{i}^{2}$ is the asymptotic variance. The asymptotic variance $s_{i}^{2}$ can be obtained in two ways: (1) $s_{i}^{2}={\left[SI/\left(2\;\times \;1.96\right)\right]}^{2}$, where *SI* is the 95% empirical support interval (Visscher *et al.* 1996; Lynch and Walsh 1998), and (2) $s_{i}^{2}$ can be obtained by the general formulas of Kao and Zeng (1997). For each contributed QTL, a segment of the cumulative normal distribution probability ranging from $u_{i}$ to $v_{i}$ is added to the EQF of the bin. If the bin (x,x+△) is on the right (left) side of the QTL position $p_{i}$, then we have $u_{i}=p_{i}+x$ ($u_{i}=p_{i}-x$) and $v_{i}=p_{i}+x+△\;{(v}_{i}=p_{i}-x-△$). In general, the contributed probability will be greater if the bin is closer to the LOD peak (QTL position), the bin size (△) is larger or the LOD score for a QTL is higher. Note that, for one QTL interval, our method using equation (1) assigns a fraction (the fractions of the within bins sum to one) and the *NL*-method assigns one to each of the within bins. For the QTL data from public databases, only the flanking markers are available for the QTL intervals, and the uniform distribution can be used to replace the normal distribution for computing the EQF value (Yang *et al.* 2019). The EQF value can be calculated at each bin for each single trait to produce the EQF matrix as $F={\left\{f_{tw}\right\}}_{T\times W}$. The sum over the EQF values of all traits, *i.e.*, $F_{w}=\sum_{t=1}^{T}f_{tw}$, at every bin will produce the EQF architecture of the genome (see Figures 2 or 3). A higher EQF value implies a greater expectation of localizing a QTL in the bin. A hotspot detection is claimed in the bin if its EQF value is higher than an EQF threshold that will be determined by permutation tests below.

### Trait grouping

**Figure 4:**
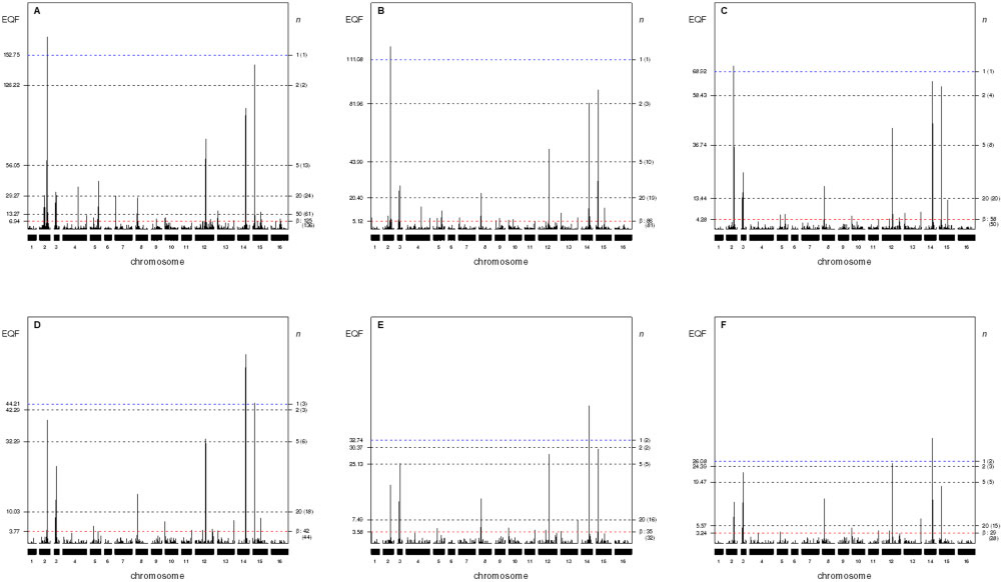
(A-F) The EQF architectures along the 16 chromosomes and the hotspots detected under different EQF thresholds ($\gamma_{n,0.05}$) at GWER of 5% in the 3- to 8-LOD EQF architectures with bin size of 2 cM for the yeast dataset. The left axis denotes the values of EQF, and the right axis denotes the values of *n*. The blue lines (first horizontal lines) correspond to the EQF thresholds $\gamma_{1,0.05}=152.75,\;111.08$, 68.92, 44.21, 32.74, and 26.08 for detecting at least one hotspot, and in practice there are 1, 1, 1, 3, 2, and 2 hotspots detected with these $\gamma_{1,0.05}s$ in the 3- to 8-LOD EQF architectures. The bottom horizontal (red) lines show the EQF thresholds of the Q-method, βs, which approximately correspond to $\gamma_{125,0.05}$, $\gamma_{86,0.05}$, $\gamma_{58,0.05}$, $\gamma_{42,0.05}$, $\gamma_{35,0.05}$, and $\gamma_{29,\;0.05},$ respectively (see text). The number in the bracket is the number of detected hotspots.

Yang *et al.* (2019) suggested grouping the genetically correlated traits together to account for the correlation structure among traits and overcome the underestimation of threshold, preventing spurious hotspots. The primary purpose of trait grouping is to group the tightly linked and/or pleiotropic traits together, and, in each trait group, the tightly linked and/or pleiotropic QTL are treated as permutation units and permuted together to cope with correlation structure among traits and obtain much stricter thresholds in detection analysis. Here, instead of using the phenotypic or genotypic correlations among traits, we use the estimated QTL positions directly to make inference about those tightly linked and/or pleiotropic traits for trait grouping. The reason is that using phenotypic or genetic correlations among traits is not effective and sufficient to group together the tightly linked and/or pleiotropic traits as shown below. Let P, G, and E denote the phenotypic value, genotypic value, and residual of a quantitative trait, respectively, then we have P=G+E as usual. For a pair of traits, $P_{1}$ and $P_{2}$, we can derive

(2) $$\rho \left(P_{1},P_{2}\right)=\rho \left(G_{1},G_{2}\right)\times \sqrt{h_{1}^{2}\times h_{2}^{2}}+\rho \left(E_{1},E_{2}\right)\times \sqrt{{(1-h}_{1}^{2})\times {(1-h}_{2}^{2})}\;$$

(see also Falconer and Mackay 1996), where $\rho \left(P_{1},P_{2}\right)$, $\rho \left(G_{1},G_{2}\right)$, and $\rho \left(E_{1},E_{2}\right)$ are the phenotypic, genetic, and residual correlations between the two traits, respectively, and $h_{i}^{2}$ is the heritability. Equation (2) tells that phenotypic correlation between two traits is an outcome of interplays among genetic correlation, residual correlation, and heritability. Some real examples of the three correlations between traits include: Zeng *et al.* (1999) analyzed the cone number and branch quality in pine and found that phenotypic correlation is very small (0.013), the genetic correlation is estimated to be significantly negative (−0.196), and the residual correlation is estimated to be significantly positive (0.189). The heritabilities are 0.560 and 0.363, respectively. We analyzed the 5,740 molecular traits in the yeast data (Brem and Kruglyak 2005) and found that there are various types of relationships between the three correlations. Figure 4 presents the phenotypic, genetic, and residual correlations of 500 randomly selected pairs of traits in the yeast data set after performing the QTL mapping analysis. It shows that, for a pair of traits, high (low) phenotypic correlation does not imply high (low) genetic correlation, and vice versa, as the heritability and residual correlation also play roles in affecting the strength of phenotypic correlation. The phenotypic and genetic correlations seem to have an arbitrary relationship, making accurate prediction of each other difficult.

We further show that a pair of tightly linked or pleiotropic traits may have arbitrary values at their genetic correlation depending on the effects and linkage parameters. First, we consider the case of two monogenic traits. Assume that the first trait, $t_{1}$, is affected by a QTL, Q*i*, and the second trait, $t_{2}$, is affected by another QTL, Q*j*, in a backcross population. We have $\rho \left(G_{1},G_{2}\right)=\pm (1\;-\;2r_{ij})$, where $r_{ij}$ is the recombination fraction between Q*i* and Q*j*, despite their effects. The value of $\rho \left(G_{1},G_{2}\right)$ is positive (negative) if they have the same (opposite) direction of effects. If the two traits are pleiotropic ($r_{ij}=0)$, we have $\rho \left(G_{1},G_{2}\right)=\pm 1$. Furthermore, we consider the case of two digenic traits. Assume that $t_{1}$ is controlled by Q*i* and Q*k*, and $t_{2}$ is controlled by Q*j* and Q_*l*__._ We have the model $G_{1}=\mu_{1}+a_{1}x_{i}$+$a_{2}x_{k}$ to model the genetic value of $t_{1}$, where $x_{i}$ and $x_{k}$ are coded variables for Q*i* and Q*k*, and $a_{1}$ and $a_{2}\;$are the effects. Similarly, we have the model $G_{2}=\mu_{2}+b_{1}x_{j}$+$b_{2}x_{l}$ to model the genetic value of $t_{2}$, where $x_{j}$ and $x_{l}$ are coded variables for Q*j* and Q*l*, and $b_{1}$ and $b_{2}\;$are the effects. For Q*i*–Q*j*–Q*k*–Q*l* order, the genetic correlation between the two traits is

(3) $$\rho \left(G_{1},G_{2}\right)=\frac{\lambda_{ij}a_{1}b_{1}+\lambda_{jk}a_{2}b_{1}+\lambda_{il}a_{1}b_{2}+\lambda_{kl}a_{2}b_{2}}{\sqrt{{[a}_{1}^{2}+a_{2}^{2}+2\lambda_{ik}a_{1}a_{2}]\times {[b}_{1}^{2}+b_{2}^{2}+2\lambda_{jl}b_{1}b_{2}]}}$$

where $\lambda_{ij}=1\;-\;2r_{ij}$ is the linkage parameter between Q*i* and Q*j*, showing that the genetic correlation is a function of QTL effects and linkage parameters. To analyze equation (3), we first consider that the four QTL are tightly linked, located in a 10-cM region and have the effects with similar magnitude but opposite sign ($a_{1}=1,a_{2}=-1,b_{1}=1,b_{2}=-1$ and $a_{1}=1,a_{2}=-1,b_{1}=-1,b_{2}=1)\;$to investigate the pattern of genetic correlations. For the case $\left(a_{1}=1,a_{2}=-1,b_{1}=1,b_{2}=-1\right)$, the genetic correlations are ρ($g_{1},g_{2})\;=0.823$ for $\left(d_{ij}=1,d_{jk}=5,d_{kl}=1\right)$, ρ($g_{1},g_{2})=0.480$ for $\left(d_{ij}=2,d_{jk}=2,d_{kl}=2\right)$, ρ($g_{1},g_{2})=0.221$ for $\left(d_{ij}=3,d_{jk}=1,d_{kl}=3\right)$, and ρ($g_{1},g_{2})=0.028$ for d.  For the case (a1=1,a2=-1,b1=-1,b2=1), the genetic correlations are ρ($g_{1},g_{2})=-0.823$ for $\left(d_{ij}=1,d_{jk}=5,d_{kl}=1\right)$, ρ($g_{1},g_{2})=-0.480$ for $\left(d_{ij}=2,d_{jk}=2,d_{kl}=2\right)$, $\rho (g_{1},g_{2})=-0.221$ for $\left(d_{ij}=3,d_{jk}=1,d_{kl}=3\right)$, and ρ($g_{1},g_{2})=-0.028$ for d. It shows that the genetic correlations between traits controlled by multiple, tightly linked QTL canhave various patterns, ranging from large (±0.823) to small values (close to 0), depending on the effects and linkage parameters. If two traits are pleiotropic for one or two QTL, the genetic correlation can be ±1 ($d_{ij}=0,d_{jk}=5,d_{kl}=0$) or close to 0 ($d_{ij}=2,d_{jk}=0,d_{kl}=2$). To simplify the analysis, we consider the case that the two QTL are pleiotropic and unlinked ($Q_{i}=Q_{j}$, $Q_{k}=Q_{l}$,$\;\lambda_{ij}=\lambda_{kl}=1$, $\lambda_{ik}=\lambda_{il}=\lambda_{jk}=\lambda_{jl}=0$) so that equation (3) reduces to

(4) $$\rho \left(G_{1},G_{2}\right)=\frac{a_{1}b_{1}+a_{2}b_{2}}{\sqrt{{\left(a_{1}b_{1}+a_{2}b_{2}\right)}^{2}+{\left(a_{1}b_{2}-a_{2}b_{1}\right)}^{2}}},\;$$

which can be considered as two different components, one ($a_{1}b_{1})\;$contributed from $Q_{i}\;=Qj$ and the other ($a_{2}b_{2})\;$contributed from $Q_{k}\;({=Q}_{l})$. The two components can be positive or negative and may sum up to any value ranging from −1 to 1 including 0. Based on equation (4), some examples of arbitrary genetic correlations between the two digenic pleiotropic traits are: If $\left(a_{1}=5,\;{\;a}_{2}=2,\;b_{1}=2,\;b_{2}=-5\right)$, we have $a_{1}b_{1}+a_{2}b_{2}=0$ and $\rho \left(G_{1},G_{2}\right)=0$. If ${(a}_{1}=2,\;a_{2}=2,\;b_{1}=2,\;b_{2}=2)$, we have $a_{1}b_{2}-a_{2}b_{1}=0$ and $\rho \left(G_{1},G_{2}\right)=1$. If ${(a}_{1}=2,\;a_{2}=2,\;b_{1}=-2,\;b_{2}=-2)$, we have $a_{1}b_{2}-a_{2}b_{1}=0$ and $\rho \left(G_{1},G_{2}\right)=-1$. If $\left(a_{1}=5,{\;a}_{2}=2,\;b_{1}=2,\;b_{2}=5\right)$, we have $a_{1}b_{1}+a_{2}b_{2}=20$ and $\rho \left(G_{1},G_{2}\right)=0.690$. If $\left(a_{1}=5,\;{\;a}_{2}=2,\;b_{1}=-2,\;b_{2}=-5\right)$, we have $a_{1}b_{1}+a_{2}b_{2}=-20$ and obtain $\rho \left(G_{1},G_{2}\right)=-0.690$. Again, depending on the relative sizes and directions of effects, their genetic correlations can be −1, −0.690, 0, 0.690, and 1, showing that the digenic pleiotropic traits may have very strong-to-very weak genetic correlations as well as no genetic correlation. If the two traits only share one pleiotropic QTL ($Q_{i}=Q_{j})$, we have $\rho \left(G_{1},G_{2}\right)=a_{1}b_{1}/\sqrt{\left(a_{1}^{2}+a_{2}^{2})(b_{1}^{2}+b_{2}^{2}\right)}$, which varies between 1 and −1 ($\left|\rho \left(G_{1},G_{2}\right)\right|<1)$ depending on the magnitude of the non-pleiotropic QTL effects ($a_{2}$ and $b_{2}$). The genetic correlation created by the pleiotropic QTL ${(Q}_{i}=Q_{j})$ is tuned (reduced) by the other non-pleiotropic QTL $Q_{k}\;\mathrm{and}\;Q_{l}$. The genetic correlation tends to be smaller, if the non-pleiotropic QTL have larger effects.

To sum up, the above analyses of equations (2–4) tell us that (1) phenotypic correlation between traits may have no relationship with their genetic correlation; (2) the genetic correlation between pleiotropic monogenic traits is either -1 or +1; (3) depending on their effect sizes and linkage parameters, the genetic correlations between closely linked or pleiotropic polygenic traits may show very weak to very strong genetic correlations, ranging between -1 and +1, including no genetic correlations, and the relationship between tightly linked or pleiotropic traits and their genetic correlations can be uncertain; (4) trait grouping based on genetic correlations can only combine the closely linked or pleiotropic traits with high genetic correlations but will fail to combine those with weak (or no) genetic correlations; and (5) grouping pleiotropic QTL together can control the correlation components contributed from themselves to account for the genetic correlations among traits. Based on the above, instead of using genetic correlations, we suggest directly using the estimated QTL positions (significant LOD peaks) to make inference about the closely linked and pleiotropic traits for trait grouping. For example, we can define those QTL being localized in the same bins (*e.g.*, bin size of 0.5, 1, or 2 cM) as tightly linked and/or pleiotropic QTL, and group their traits together into the same trait groups (hereinafter referred to as “empirical trait grouping”). In each trait group, the tightly linked and/or pleiotropic QTL will be treated as a trunk or unit in permutation, and the other QTL will be permuted along to account for the correlation structure among traits and compute stricter thresholds for assessing the significance of QTL hotspots.

### Permutation algorithm for computing the EQF thresholds $\gamma_{n,\alpha }$’s

We devise two permutation schemes, the QTL-interval permutation and the EQF-bin permutation, to compute the EQF thresholds for assessing the significance of QTL hotspots. The QTL-interval permutation operates on the QTL matrix and then considers the genome to be circular (Cabrera *et al.* 2012) and randomly swaps the QTL intervals in the circular genome. On the contrary, the EQF-bin permutation works on the EQF matrix and then breaks a QTL interval into several EQF bins and randomly shifts the EQF bins along the genome. When trait grouping is considered to cope with the correlation structure among traits, the QTL intervals or EQF bins associated with the tightly linked and/or pleiotropic QTL will be permuted together across the genome separately for each trait group to obtain stricter EQF thresholds. Below the QTL-interval permutation algorithm is first outlined, and the EQF-bin permutation algorithm will follow without much difficulty.

Using the *m* different LOD thresholds, we have converted the LOD score matrix into the *m* QTL matrices and then *m* EQF matrices, respectively. For an EQF matrix, F, we first obtain the EQF sum over all traits for the *W* bins and order them from highest to lowest, $F_{\left(1\right)}$, $F_{\left(2\right)}$, …, $F_{\left(W\right)}$. Then, we define qFreq(n) as the *n*th EQF sum of the *W* ordered observed EQF sums and use qFreq(n) as a test statistic for at least *n* spurious hotspots under the null hypothesis that the QTL are randomly distributed in the genome. The algorithm of the QTL-interval permutation with trait grouping for computing the threshold that can control GWER of $\text{qFreq}\left(n\right)$ at a fixed α level is described below:

1. The traits with tightly linked or pleiotropic QTL are grouped together. After grouping, there are, say *R*, trait groups containing $g_{1},\;g_{2},\;g_{3},\;\ldots ,\;g_{R}\;\;$ traits, respectively $\left(\sum g_{i}=T\right)$.
2. Generate a new permuted QTL matrix by performing permutation in each trait group as follows: first consider the genome to be circular. For each trait group, the QTL intervals of the tightly linked or pleiotropic QTL are permuted together and the other QTL intervals are permuted alone to obtain a permuted QTL matrix.
3. Obtain the EQF matrix, $F^{*},$ from the permuted QTL matrix,and compute the total EQF sums over all rows for the *W* locations in, *i.e*. ${\;F}_{w}^{*}=\sum_{t=1}^{T}f_{\;tw}^{*}\;\mathrm{for}\;w=1,\;2,\;\ldots ,\;W.$ for w=1,2,…,*W*. Then,order the *W* EQF sums $\;(F_{w}^{*}\prime s)$ from highest to lowest as $\;F_{\left(1\right)}^{*},\;F_{\left(2\right)}^{*},\;\ldots ,\;F_{\left(W\right)}^{*}$
4. For a fixed hotspot number *n*, obtain and store $F_{\left(n\right)}^{*}\;$. corresponding to the *n*th EQF sum of the *W* ordered EQF sums for $F^{*}$
5. Repeat steps 1–4 B  times so that there are *B* new permuted matrices $\;(\mathrm{namely},\;F^{1},\;F^{2},\ldots ,\;F^{B})$ for obtaining the $\;F_{\left(n\right)}^{1},\;F_{\left(n\right)}^{2},\ldots ,\;F_{\left(n\right)}^{B}.\;$ The *B*--permutation samples of $\;F_{\left(n\right)}^{i},\;i=1,\;2,\;\ldots ,\;B,$ are the estimate of the null distribution of the test statistic $\text{qFreq}\left(n\right)\;$for at least *n* spurious hotspots anywhere in the genome under the null.
6. The upper (1-α), -quantile of the B-permutation samples generated in step 5 is the EQF threshold,denoted by $\gamma_{n,\alpha }$ for $\text{qFreq}\left(n\right)$ for assessing at least *n* spurious hotspots.

If the EQF-bin permutation with trait grouping is considered, the permutation is performed on the original EQF matrix, F, directly. In steps 2 and 3, for each trait group, the EQF bins associated with the tightly linked or pleiotropic QTL are permuted together and those of the other QTL intervals are permuted alone to obtain a permuted EQF matrix, $F^{*}$ (see also Yang *et al.* 2019). Then, we order the *W* EQF sums ($F_{w}^{*}s)\;$from highest to lowest as$\;F_{\left(1\right)}^{*}$, $F_{\left(2\right)}^{*}$, …,$\;F_{\left(W\right)}^{*}$ to obtain the $\gamma_{n,\alpha }$ threshold by using step 4 to step 6. In this way, the proposed algorithm can deploy both the EQF-bin permutation and the QTL-interval permutation to compute a series of thresholds, $\gamma_{n,\alpha }$s, for $\text{qFreq}\left(n\right)$s to assess the significance of QTL hotspots. For n=1, 2, …, *k*, where *k* is determined by $\beta =\gamma_{k,\alpha }$ (*β* is the threshold value obtained without trait grouping, *i.e.*, by using the Q-method), a series of $\gamma_{n,\alpha }$s ranging from the most conservative (n=1) to the most liberal (n=k) can be obtained and used for assessing the significance of different numbers of QTL hotspots. By adopting $\gamma_{n,\alpha }$, it can control GWER of $\text{qFreq}\left(n\right)$ at level α of detecting at least *n* spurious hotspots under the null, as detecting more than *n* hotspots is less likely than detecting *n* hotspots given the threshold $\gamma_{n,\alpha }$. Since the permutation algorithm is performed on the QTL matrix or EQF matrix and only one QTL mapping analysis is required in the whole procedure, our framework has a very cheap computational cost as compared to the *N*-method and *NL*-method that need to perform many repeated QTL mapping analyses, and hence is suitable for practical use.

### The top $\gamma_{n,\alpha }$ profile

**Figure 5:**
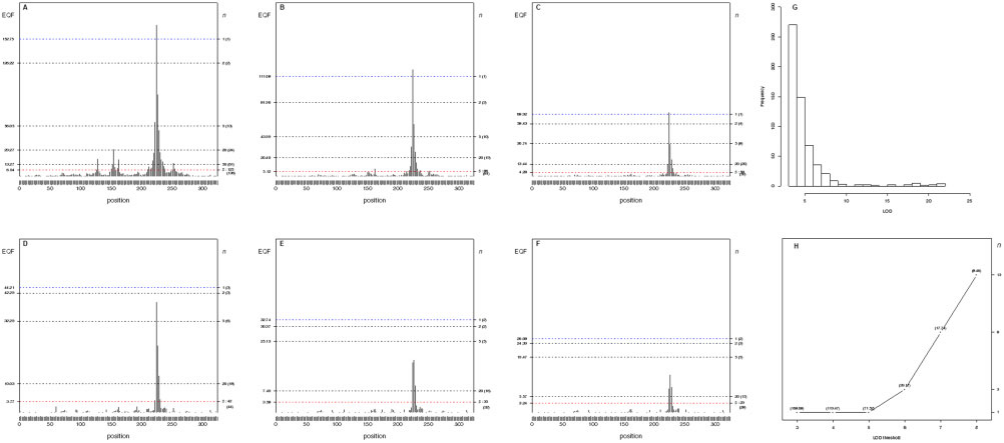
(A–H) The 3- to 8-LOD EQF architectures of the second chromosome and the $\gamma_{n,0.05}$ EQF thresholds at GWER of 5% for the yeast dataset. The left axis denotes the values of EQF, and the right axis denotes the values of *n*. (A–F) The peak at the bin [2,224] is significant as a hotspot under the $\gamma_{1,0.05}\left(3\right)=152.75,\;\;\gamma_{1,0.05}\left(4\right)=111.08,\;\;\gamma_{1,0.05}\left(5\right)=68.92,\;{\;\gamma }_{3,0.05}\left(6\right)=38.66,\;\;\gamma_{8,0.05}\left(7\right)=16.70$, and $\gamma_{13,0.05}\left(8\right)=8.29$ thresholds in the 3- to 8-LOD EQF architectures. For (A)–(F), the number in the bracket is the number of detected hotspots. (G) The distribution of LOD scores >3 for the QTL at the bin [2,224]. (H) The top $\gamma_{n,0.05}$ profile shows that the values of *n* are 1, 1, 1, 3, 8, and 13, showing an increasing pattern, across the 3- to 8-LOD EQF architectures. For (G) and (H), the number in the bracket is the EQF value of the bin.

The permutation algorithm allows to compute a series of EQF thresholds, $\gamma_{n,\alpha }$s, for each of the *m* EQF matrices. Now, we denote $F\left(L_{i}\right)$ as the EQF matrix constructed using LOD threshold $L_{i}$ and $\gamma_{n,\alpha }(L_{i})$s as the corresponding thresholds for $F\left(L_{i}\right)$. We define the top $\gamma_{n,\alpha }(L_{i})$ threshold for a bin *w* as

(5) $$\mathrm{Top}\;\gamma_{n,\alpha }\left(L_{i}\right)=\;\underset{n}{\min}{\{\gamma }_{n,\alpha }(L_{i})\}\leq F_{w}(L_{i}),$$

where${\;F}_{w}(L_{i})$ is the EQF sum of the bin *w* over all traits in the $F\left(L_{i}\right)$ matrix. That is, the top $\gamma_{n,\alpha }(L_{i})$ threshold is the largest EQF threshold (with the smallest *n*) for bin *w* to be significant as a QTL hotspot in the $F\left(L_{i}\right)$ matrix. For a hotspot, the smaller the value of *n* in the top $\gamma_{n,\alpha }$ threshold is, the relatively more significant it is. Therefore, in a specific EQF architecture, the top $\gamma_{n,\alpha }$ threshold of a hotspot can be used to characterize its significance status compared to the other hotspots. For a hotspot, there are *m* top $\gamma_{n,\alpha }$ thresholds across all the *m* EQF architectures. The pattern of (the *n* values in) the *m* top $\gamma_{n,\alpha }$ thresholds can outline how the relative significance status of a hotspot changes over the different EQF architectures. For example, in Figure 1B, the hotspot (hotspot A) has an EQF value 55 in $F\left(2\right)$ and an EQF value 53 in $F\left(6\right)$. The top $\gamma_{n,\alpha }$ thresholds are $\gamma_{3,0.05}\left(2\right)$ and $\gamma_{2,0.05}\left(6\right)$ with values of *n* being 3 and 2, respectively, meaning that the hotspot has a relatively large amount of QTL with large LOD scores (LOD scores >6) as compared to other bins. In Figure 1D, the hotspot (hotspot C) has an EQF value 71 in $F\left(2\right)$ and an EQF value 13 in $F\left(6\right)$. The top $\gamma_{n,\alpha }$ thresholds are $\gamma_{2,0.05}\left(2\right)$ and $\gamma_{7,0.05}\left(6\right)$ with values of *n* being 2 and 7, respectively, meaning that the hotspot has a relatively less amount of QTL with large LOD scores (LOD scores >6) as compared to other bins. Therefore, by investigating how the value of *n* changes among the top $\gamma_{n,\alpha }(L_{i})$ thresholds of a hotspot, we can understand the LOD-score distribution of a hotspot relative to those of other hotspots across the different EQF architectures. In this way, we can compute and profile the top $\gamma_{n,\alpha }$ thresholds for a hotspot in different $F\left(L_{i}\right)$ matrices (say $L_{i}=3,\;4,\;5,\;6,\;7,\;8$, depending on the number of trait and genome size), and further use the top $\gamma_{n,\alpha }$ profile to outline the LOD-score distribution of QTL in a hotspot. If the top $\gamma_{n,\alpha }$ profile of a hotspot shows a decreasing (increasing) pattern of *n* with $L_{i}$ increasing, we may conclude that the hotspot contains relatively more (fewer) QTL with high LOD scores, as compared to other hotspots (see Figures 1 and 5). If the profile has a nearly flat pattern, we may infer that the QTL in the hotspot are well-balanced in the magnitude of the LOD scores. Based on the above interpretation, the pattern of top $\gamma_{n,\alpha }$ profile of a QTL hotspot can readily identify and characterize the types of hotspots with varying sizes and LOD-score distributions.

### Data availability

All data and the R codes for the data analysis used in this article are available at http://www.stat.sinica.edu.tw/chkao/.

Supplementary material is available at https://doi.org/10.25387/g3.13614122.

## Results

In this section, simulation study and real example analysis are conducted to illustrate the proposed statistical framework, investigate the related properties, and evaluate the performance as well as compare with the current methods in QTL hotspot detection. In simulation study, we investigate the performance of the proposed statistical framework and compare with the *Q*-method, the *N*-method, and the *NL*-method in detecting QTL hotspots. In real example analysis, we first apply the statistical framework to analyze the summarized QTL data collected in GRAMENE rice database (Yang *et al.* 2019) and then to analyze the individual-level yeast data set in Brem and Kruglyak (2005) and compare the results with those in Yang *et al.* (2019) and Neto *et al.* (2012) in QTL hotspot detection, respectively. We also investigate and validate the patterns of genetic correlations among closely linked and pleiotropic traits in the yeast data set to confirm the theoretical analysis in *trait grouping*.

### Simulation study

Yang *et al.* (2019) have performed the simulation study to show that the permutation procedure with trait grouping can control GWERs at the target levels for the QTL data with correlation structure and has the ability to produce quality results by offering a sliding scale of thresholds for QTL hotspot detection. In the Yang *et al.* simulation study, all traits were assumed to be monogenic, and a perfect trait grouping, in which all the simulated pleiotropic traits were correctly grouped together, is considered for grouping traits. Here, in addition to considering multigenic traits, we further show the effectiveness of empirical trait grouping, in which the traits with QTL being localized in the same bins are grouped together, and investigate the ability of top $\gamma_{n,\alpha }$ profile in characterizing and identifying the different types of QTL hotspots with varying sizes and LOD-score distributions in the detection analysis. Likewise, we simulate a small-scale genetical genomics data set that contains 100 backcross progeny with 5 chromosomes of length 100 cM and 600 molecular traits. Each chromosome contains 50 equally spaced markers, and the bin size of 2 cM (similar to that in Yang *et al.* 2019) is used in the analysis. The 600 traits are assumed to be monogenic or multigenic. Three unlinked hotspots A, B, and C are considered: (1) a small hotspot A is caused by a gene at 50 cM of the first chromosome and affects 100 pleiotropic traits with heritabilities 0.3–0.45 showing high LOD scores in QTL mapping; among the 100 pleiotropic traits, we assume that 60 traits are monogenic, and the other 40 traits are digenic or trigenic and also affected by other genes located in the second and/or fourth chromosome. (2) A big hotspot B is caused by a gene located at 50 cM of the third chromosome and contains 300 pleiotropic traits. Among the 300 pleiotropic traits, half have heritabilities 0.1–0.45 showing moderate-to-high LOD scores, and half have heritaibilities 0.3–0.45 showing high LOD scores. (3) A big hotspot C contains 200 traits and is caused by a gene at 50 cM of the fifth chromosome. The heritabilities of the 200 traits are 0.1–0.2 showing small LOD scores. Supplementary Figure S1, A–D, shows the LOD distributions of the traits in the three hotspots and the distribution of the pairwise correlation among traits for the simulated data set. The pairwise correlations among traits vary from −0.42 to 0.67 with mean 0.114 (Supplementary Figure S1D). For the purpose of comparison, the bin containing the estimated QTL position will be given to 1 (and 0 otherwise) to construct the QTL matrix for the operation of the *Q*-method and our approach. The EQF-bin permutation and empirical trait grouping are adopted in the analysis. We also adopt 1.5-LOD 95% support intervals to decrease the spread of the hotspots for the *N*-method and *NL*-method (Neto *et al.* 2012).

**Figure 6:**
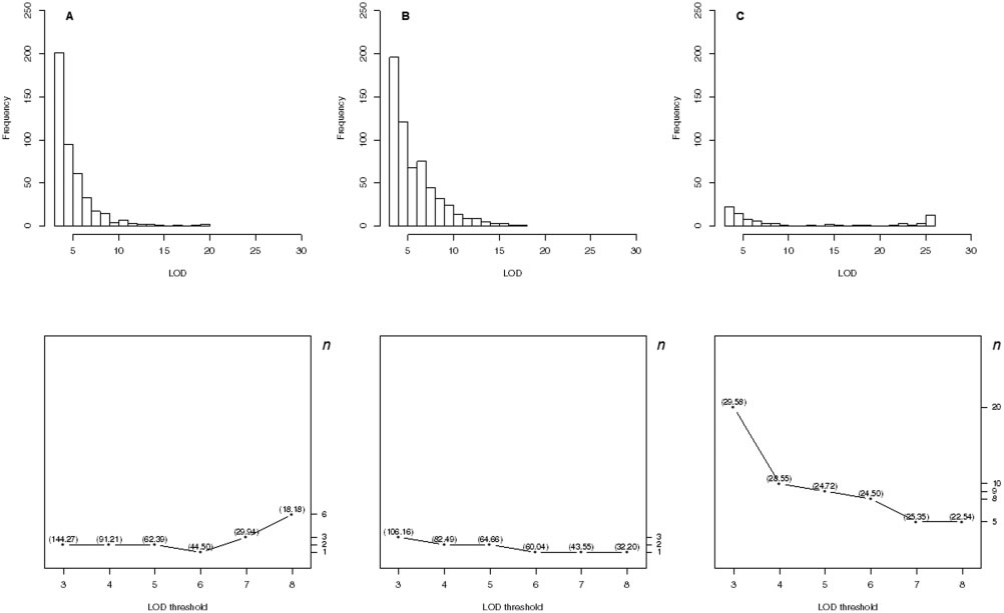
(A–C) The distributions of QTL with LOD scores >3 and the top $\gamma_{n,0.05}$ profiles for the bins [15,60], [14,242] and [3,89.5] (the first marker on chromosome 3 starting at the position of 9.5 cM) for the yeast dataset. The upper panels display the distributions of LOD scores, and the bottom panels show the top $\gamma_{n,0.05}$ profiles. (A) The top $\gamma_{n,0.05}$ profile displays that the *n* values have an increase trend within the narrow range from 1 to 5 over the 3- to 8-LOD thresholds, showing that the bin [15,60] is a major hotspot containing QTL with balanced LOD scores. (B) The top $\gamma_{n,0.05}$ profile displays a flat pattern with the values of *n* varying within the range of from 1 to 3 showing that the bin [14,242] is also a major hotspot containing the QTL with balanced LOD scores. (C) The top $\gamma_{n,0.05}$ profile has a decreasing trend over the 3- to 8-LOD thresholds, showing that the bin [3,89.5] is also a major hotspot containing relatively more QTL with ≥6 LOD scores. The number in the bracket is the EQF value of the bin.

The results of the *Q*-method, *N*-method, the *NL*-method, and the proposed procedure (with empirical trait grouping) are summarized in Supplementary Figure S2, A–F, which are similar to those in Figure 6 of the Yang *et al.* paper using perfect trait grouping. Supplementary Figure S2A and Figure 1 present the hotspot architecture constructed using a single-trait LOD threshold of 2.47 (obtained by permutation test) and the 5% significance hotspot size thresholds obtained by the *Q*-method, *N*-method, and proposed frameworks. The hotspots on the first, third, and fifth chromosomes have sizes 55, 214, and 67, respectively. The hotspot size thresholds obtained by the *Q*-method and *N*-method are 3 and 16, which correspond to${\;\gamma }_{21,0.05}$ and ${\;\gamma }_{13,0.05}$ by our approach. Supplementary Figure S2, B–F, present the hotspot architectures inferred using the *NL*-method LOD thresholds of 5.19, 3.77, 1.58, 1.36, and 1.13 that aim to control GWER of 5% for spurious hotspots of sizes 1, 3, 43, 60, and 90, respectively. It shows that, in addition to detecting the three true hotspots, the *Q*-method and *N*-method also detect several spurious hotspots (20 and 9 in total) near the true hotspots due to lower thresholds, and the proposed procedure can produce less spurious hotspots due to higher thresholds (see Figure 1A). For example, using${\;\gamma }_{2,0.05}=76$ (the threshold for detecting at least two hotspots) as a threshold, only the hotspot on the third chromosome is detected (Figure 1A). Using${\;\gamma }_{4,0.05}=36$ (the threshold for detecting at least four hotspots) as a threshold, all the three hotspots can be detected (Figure 1A). It shows that empirical trait grouping in our framework is effective to cope with the correlation structure among traits and can obtain stricter thresholds, preventing spurious hotspots in QTL hotspot detection. Figure 1, B–D, presents the top $\gamma_{n,\alpha }$ profiles of the three hotspots, A, B, and C, in the 2- to 6-LOD EQF architectures. In Figure 1B, the top $\gamma_{n,\alpha }$ profile of hotspot A has a decreasing pattern with the value of *n* decreasing from 3 to 2 over the 2- to 6-LOD EQF architectures, indicating that hotspot A contains relatively more QTL with large LOD scores. In Figure 1C, the top $\gamma_{n,\alpha }$ profile for hotspot B has a flat pattern with *n *=* *1 for all the EQF architectures, indicating that hotspot B is a major hotspot containing QTL with balanced LOD scores. In Figure 1D, the top $\gamma_{n,\alpha }$ profile of hotspot C shows an increasing pattern with the value of *n* increasing from 2 to 7 over the five EQF architectures, indicating that hotspot C contains relatively fewer QTL with large LOD scores. Therefore, the patterns of top $\gamma_{n,\alpha }$ profiles can outline the LOD-score distributions of the hotspots, A, B, and C, well. To sum up, the simulation study shows that the proposed statistical procedure with trait grouping and top $\gamma_{n,\alpha }$ profile has the ability to produce quality results by offering a sliding scale of thresholds from high to low for QTL hotspot detection and is applicable to distinguish the different types of hotspots in the hotspot analysis.

### The GRAMENE rice dataset

The GRAMENE database is a web-accessible and common reference database for crop research. For rice, it collects 8,216 QTL (*N *=* *8,216) responsible for 236 different traits (*T *=* *236) from 230 published studies (experiments). The total length of the rice 12 chromosomes is ∼1,536.9 cM. There are 1,914 common markers on the consensus map with an average marker density of one marker every 0.81 cM. The QTL density is ∼5.35 QTL per cM. Among the 8,216 QTL collected in the GRAMENE database, 309 (3.76%) QTL are localized at markers, 3,791 (46.14%) QTL are localized in the marker intervals with sizes between 0 and 0.5 cM, 74 (0.90%) QTL are localized in the 0.5-1 cM intervals, 200 (2.43%) QTL are localized in the 1–2 cM intervals, 509 (6.20%) QTL are in the 2–5 cM intervals, 6.94 (5.57%) QTL are in the 5–10 cM intervals, and 1,023 (12.45%) QTL are in the 10–20 cM intervals. The medium, mean, and SD of the interval sizes are 0.56, 9.82, and 16.82 cM, respectively. It implies that, if bins are identified as hotspots, the major contribution to their EQF is from the ≦1 cM QTL intervals and that the large QTL intervals only contribute a small portion to the EQF of the hotspots. The flanking marker pairs of the 8,216 QTL (8,216 QTL intervals) are recorded and used for detecting QTL hotspots. By using equation (1) with uniform distribution and using bin size of 0.5 cM (△=0.5 cM), the EQF architectures (Figure 2) and the EQF matrix (with a dimension 236 × 3,070) can be obtained. When adopting empirical trait grouping in permutation analysis, only those QTL intervals ≦0.5 cM are considered, and there are a total of 71 trait groups in the analysis.

Figure 2 presents the EQF architectures of the 12 chromosomes and the hotspots detected under different EQF thresholds. In Figure 2, the first highest peak (on the 4th chromosome) with EQF value 71.97 is significant under the threshold $\gamma_{1,0.05}=71.81$ Under $\gamma_{3,0.05}=$56.55, there are two hotspots detected (on the third and fourth chromosomes). The highest peak of the first chromosome has an EQF value 35.89 and was significant under $\gamma_{10,0.05}=$35.88, but not significant under $\gamma_{9,0.05}=36.06$, and the top $\gamma_{n,\alpha }$ threshold associated with this peak is $\gamma_{10,0.05}$. Under $\gamma_{9,0.05}=36.06$, there are 7 significant hotspots in practice. Under $\gamma_{100,0.05}$, there are 102 significant hotspots (not shown). Chardon *et al.* (2004) empirically suggested five times of the average EQF value per bin (5.35 ÷ 2 × 5 = 13.38) as the threshold, which roughly corresponds to $\gamma_{109,0.05}=$13.34. Under $\gamma_{109,0.05}$, there are 116 significant hotspots detected (not shown). The EQF threshold obtained by the *Q*-method is about 9.75 (corresponding to $\gamma_{179,0.05}$, where 179 is the upper bound of *n*, *i.e.*, *k *=* *179), leading to the detection of 179 QTL hotspots, among which many of them are believed to be spurious since the EQF threshold obtained by the *Q*-method is too liberal (Neto *et al.* 2012; Yang *et al.* 2019). As compared to the result of Figure 4 in Yang *et al.* (2019) using trait grouping based on the general agronomic consideration (with nine trait groups), the EQF thresholds obtained by empirical trait grouping (with 71 trait groups) are higher, and the observed and expected hotspot numbers are closer to each other.

### The yeast dataset

The yeast data consist of expression measurements on 5,740 transcripts and 2,956 genetic markers on 112 segregant strains (Brem and Kruglyak 2005). The expression measurements are further converted to normal quantiles for the hotspot analysis as described by Neto *et al.* (2012) and are available in the R package yeastqtl (https://github.com/byandell/qtlyeast). Among the 2,956 markers, numerous markers are in complete linkage disequilibrium, and only one of them will be used in the analysis. In total, 1,072 markers are used in the analysis. The genome size is ∼6,345 cM. The average marker density is about one marker every ∼5.92 cM. For comparison with the QTL hotspot detection analysis in Neto *et al.* (2012), the QTL mapping analysis was performed also using the regression interval mapping (Haley and Knott 1992) with the same bin size of 2 cM (Δ=2 cM) under a single-QTL model with the R/qtl software (Broman *et al.* 2003). The LOD scores at all bins for each trait were recorded to construct the LOD score matrix, and the 1.5-LOD 95% support interval (Neto *et al.* 2012) is used to construct the EQF matrix for the analyses that follow. The EQF-bin permutation with empirical trait grouping is performed to obtain the EQF thresholds.

For the same yeast data set, Neto *et al.* (2012) used a single-trait permutation LOD threshold of 3.45 corresponding to a 5% GWER to claim the QTL detection and construct the QTL matrix and obtained 7.40 as the conservative LOD threshold (associated with hotspot size 1). Using the Gaussian process (Guo 2011), the relaxed LOD threshold for one trait ranges from ∼3.05 to ∼3.48, and the conservative LOD threshold for 5,740 traits is ∼6.17, respectively. We therefore use a sliding scale of empirical LOD thresholds of 3, 4, 5, 6, 7, and 8 for claiming QTL detection and constructing the six corresponding QTL matrices. Using the six LOD thresholds, among the 5,740 molecular traits, there are 5,586, 2,797, 1,740, 1,232, 911, and 696 QTL detected for 3,863, 2,455, 1,656, 1,205, 895, and 688 traits, respectively, among which multiple QTL were detected for 1,402, 325, 83, 27, 16, and 8 traits, respectively. As it should be, higher LOD thresholds will result in fewer detected QTL but with larger LOD scores. The QTL densities are less than one QTL per cM (∼0.88, ∼0.44, ∼0.26, ∼0.19, ∼0.14, and ∼0.11 under the six different LOD thresholds) for the six QTL matrices. The empirical trait grouping results in a total of 475, 562, 523, 450, 375, and 308 trait groups under the six different LOD thresholds.

Figure 3, A–F, presents the 3- to 8-LOD EQF architectures of the yeast genome and the hotspots detected under different EQF thresholds (at 5% GWER). In Figure 3, A–F, the threshold values, $\gamma_{n,\alpha }s$, for the test statistic qFreq(*n*)s are coordinately represented by the left and right axes. For example, in the 3-LOD EQF architecture, the first highest EQF peak is located in the bin [2,224] (223–225 cM of the second chromosome), which is significant under the threshold $\gamma_{1,0.05}\left(3\right)=152.75$ for at least one spurious hotspot. The bin [2,224] is also the highest EQF peak in the 4- and 5-LOD EQF architectures, and is the 4th, 10th and 14th highest peak, respectively, in the 6- to 8-LOD EQF architectures. Under the six $\gamma_{1,0.05}$ thresholds, the bin [2,224] is significant in the 3- to 5-LOD EQF architectures, but not significant in the 6- to 8-LOD EQF architectures. There are 1, 1, 1, 3, 2, and 2 bins significant as hotspots with the six $\gamma_{1,0.05}$ thresholds, respectively. Similarly, under the $\gamma_{5,0.05}$ thresholds, there are 13, 10, 8, 6, 5 and 5 bins significant as hotspots in the six EQF architectures. The EQF thresholds obtained by the *Q*-method (βs) correspond to about $\gamma_{125,0.05}$, $\gamma_{86,0.05}$, $\gamma_{58,0.05}$, $\gamma_{42,0.05}$, $\gamma_{35,0.05}$, and $\gamma_{29,0.05}$, respectively, for the 3- to 8-LOD EQF architectures. Using these βs, there are 136, 81, 50, 44, 32, and 28 significant bins, among which most of them are believed to be spurious since the βs are known to be too liberal due to ignoring the correlation structure among traits, in the six EQF architectures. In general, Figure 3, A–F, displays several obvious peaks, indicating that there must exist several hotpots in the yeast genome. More detailed chromosome-by-chromosome plots are presented in Supplementary Figure S3, 1–21.

Figure 6, A–H, presents the six EQF architectures of the bin [2,224] on the second chromosome, its top $\gamma_{n,\alpha }$ profiles and distribution of QTL with LOD scores >3. Figure 6H displays that the top $\gamma_{n,\alpha }$ thresholds of the bin [2,224] are $\gamma_{2,0.05}(3)$, $\gamma_{2,0.05}(4)$, $\gamma_{1,0.05}(5)$, $\gamma_{3,0.05}(6)$, $\gamma_{8,0.05}(7)$ and $\gamma_{13,0.05}\left(8\right)$ across the six EQF architectures. The values of *n* increase from 2 to 13 over the six LOD thresholds, meaning that the bin [2, 224] is more significant as a hotspot by using the <6 LOD thresholds as compared to by using the ≥6 LOD thresholds and, therefore, can be regarded as a major QTL hotspot containing relatively more QTL with <6 LOD scores (moderate LOD scores) and relatively fewer QTL with ≥6 LOD scores (large LOD scores). Figure 6G shows the distribution of LOD scores, which is more abundant in QTL with moderate LOD scores. Figure 5, A–C, displays the distributions of LOD scores (upper panels) and the top $\gamma_{n,0.05}$ profiles (bottom panels) for the 2nd, 3^rd^, and 24th highest peaks at [15,60], [14,242], and [3,89.5], respectively. The three top $\gamma_{n,0.05}$ profiles have a slightly increasing trend (within the narrow range of 1 to 6), a near flat pattern, and a decreasing pattern over the EQF architectures, respectively. It tells that both the bin [15,60] and bin [14,242] are major hotspots containing QTL with balanced LOD scores (moderate-to-large LOD scores) and that the bin [3,89.5] is also a major hotspot containing relatively more QTL with larger LOD scores, as can be also perceived in the distributions of LOD scores (upper panels). The EQF architectures, the top $\gamma_{n,\alpha }$ profiles, and distributions of LOD scores of the 23 significant peaks are placed in Supplementary Figure S3, 1–21. In general, the significance status of a bin as a hotspot and the types of the hotspots can be well characterized by using the top $\gamma_{n,\alpha }$ profile.

We also compared the above results obtained by the proposed statistical framework with those by the *Q*-, *N*-, and *NL*-methods presented in Neto *et al.* (2012). The *Q*-method produces liberal thresholds and detects many spurious hotspots (see above). The *N*-method detected five major hotspots on chromosomes 2, 3, 12, 14, and 15, and a suggestive hotspot (almost reaches significance) on chromosome 8, which are also detected by the *NL*-method (Neto *et al.* 2012) and by our statistical framework (correspond to the hotspots at the bins [2,224], [3,89.5], [8,60], [12,324], [14,242], and [15,60] in our analysis). Notably, our framework detects two hotspots at [3,61] and [3,89] on the third chromosome. Other small peaks on chromosomes 1, 4, 5, 7, 9, 13, and 16 considered by the *NL*-method are also detectable with our statistical framework (using less strict thresholds). Both the *NL*-method and our framework can assess the significance of hotspots with any type of LOD-score distribution. We follow Neto *et al.* to classify the hotspots into three types: (i) a hotspot composed of many QTL with moderate (<6) LOD, (ii) a hotspot consisting of a few QTL with strong (≧6) LOD scores, and (iii) a large hotspot containing QTL with a range of moderate-to-large LOD scores. In our statistical framework, the top $\gamma_{n,\alpha }$ profiles for the type i, ii, and iii hotspots will respectively show increasing, decreasing and flat patterns. The *NL*-method summarized that the hotspots on chromosomes 2, 3, 12, 14, and 15 are of type iii, the hotspots on the 5th, 8th, and 13th chromosomes are of type ii, and no hotspot is of type i. Using the top $\gamma_{n,\alpha }$ profiles, our framework concludes that the hotspots at bin [2,224] and bin [5,258] on the second and fifth chromosomes can be considered as being of type i (see Figure 3 and Supplementary Figures S3-7), the hotspot at bin [3,89.5] on the third chromosome is of type ii, not type iii by the *NL*-method, and the hotspots on the 12th, 14th, and 15th chromosomes are of the same type (type iii) as the *NL*-method (see Supplementary Figures S3-16). Also, the hotspots at bin [8,60] on the 8th chromosome and at bin [13, 516] on the 13th chromosome are of the same type (type ii) as the *NL*-method (Supplementary Figures S3-12 and S3-18). Neto *et al.* identified two significant peaks on each of the 2nd, 12th, and 15th chromosomes; Nevertheless, we only observed a single significant peak on each of them but detected two peaks (at bins [3,57.5] and [3,89.5]) on the third chromosome. The other small peaks at bins [4,464] and [7,30] on the fourth and seventh chromosomes are less interesting but may be classified as type i according to their top $\gamma_{n,\alpha }$ profiles (see Supplementary Figures S3-5 and S3-10). In general, the top $\gamma_{n,\alpha }$ profile can be used to characterize the three types of hotspots, and the results by the *NL-*method and our statistical framework are conformable in the detection and classification of QTL hotspots.

### The pairwise phenotypic and genetic correlations in trait groups

**Figure 7:**
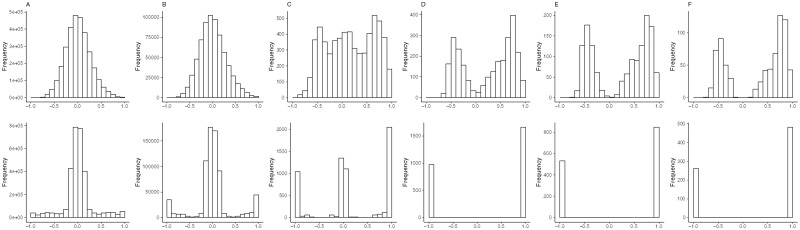
(A–F) The distributions of pairwise phenotypic and genetic correlations in the first largest trait groups (containing most traits) of the 3- to 8-LOD EQF architectures for the yeast dataset. (A–F) The largest trait groups contain 3,124, 1,411, 136, 78, 55, and 40 pleiotropic traits (pleiotropic QTL), among which there are 1,299, 221, 5, 0, 0, and 0 traits are detected with multiple QTL, respectively, in the six EQF architectures. The upper and bottom panels show the pairwise phenotypic and genetic correlations, respectively.

We show by equations (2–4) that trait grouping based on the phenotypic or genetic correlations is not effective in combining closely linked or pleiotropic traits. Also, genetic correlations between monogenic pleiotropic traits are either -1 or +1, and genetic correlations between multigenic pleiotropic traits can be arbitrary values between -1 and +1 (including zero), depending on the relative sizes and directions of effects and their linkage parameters. The above argument can be also justified by analyzing the pairwise phenotypic and genetic correlations among traits (with closely linked or pleiotropic QTL) in the same trait group in the yeast data. Figure 7, A–F, displays the distributions for all pairwise phenotypic and genetic correlations among the traits in the first largest trait groups (the bin containing the most traits) in the 3- to 8-LOD EQF architectures. The largest trait groups contain 3,124, 1,411, 136, 78, 55, and 40 pleiotropic traits, among which there are 1,299, 221, 5, 0, 0, and 0 traits are detected with multiple QTL, respectively, in the six EQF architectures. In the case of the 3-LOD EQF architecture (Figure 7A), as many (1,297) pleiotropic traits are multigenic, their pairwise genetic correlations vary between +1 and -1, with a large proportion falling between -0.25 and 0.25. When the LOD thresholds become stricter, it will produce less detected QTL, less multigenic, and more monogenic pleiotropic (or linked) traits (in proportion), causing that pairwise genetic correlations of -1 or +1 gradually become the most dominant feature (Figure 7, A–F, bottom panels). In Figure 7, D–F (bottom panels), the cases of the 6-LOD, 7-LOD, and 8-LOD EQF architectures, all the 78, 55, and 40 traits are monogenic and hence the pairwise genetic correlations among them are either -1 or +1 (see subsection *trait grouping*). The distributions for the pairwise phenotypic correlations among traits in the largest trait group are also presented in Figure 7, A–F (upper panels), showing that the pleiotropic traits have a vaguer relationship with their phenotypic correlations. The distribution of pairwise phenotypic correlations shows a bell shape in the cases of 3- and 4-LOD EQF architectures (Figure 7, A and B upper panels), becomes a plateau type in the case of 4-LOD EQF architecture (Figure 7C, upper panels), and has a bimodal pattern in the cases of 6-, 7- and 8-LOD EQF architectures (Figure 7, D–F upper panels), respectively. In general, the results in Figure 7 and the analytical derivations in equations (2–4) are compatible and confirm each other. The results also validate the use of the estimated QTL positions rather than the phenotypic or genetic correlations for trait grouping that intends to take into account the linkages among QTL for avoiding spurious hotspots in our statistical framework.

## Discussion

Genome-wide detection of QTL hotspots requires first to collect the data with many QTL widespread in the genome and, then, to construct the hotspot architectures and further to determine the thresholds for assessing the significance of QTL hotspots. The public databases and genetical genomics studies are two feasible ways to provide the data containing many QTL. The public databases curate abundant summarized QTL data for various traditional traits from numerous published studies (experiments), and the genetical genomics study can produce adequate individual-level data for many molecular traits in a single study for the detection of QTL hotspots. The GRAMENE rice database collects QTL for many traditional traits from numerous independent experiments. It is likely that the same traits are investigated and mapped for the same QTL in different experiments. Ideally, these same QTL (reproducible QTL) should only be counted once in counting process to avoid over counts of QTL for hotspot sizes. Otherwise, there is a concern about over counts of QTL, leading to artifactual hotspots, in the analysis. The genetical genomics study allows to perform the permutation analysis on both the QTL matrix and individual-level data, and the public database to date allows the analysis only on the QTL matrix to obtain the significance thresholds. Notably, permuting the QTL matrix has the outstanding advantage of very low computational cost, and permuting the individual-level data has the benefit of preserving the correlation structure among traits but comes with very expensive computational cost (see *Introduction*). Our statistical framework is deployed on the QTL matrices and hence can deal with both types of data with very low computational effort for QTL hotspot detection. Also, we introduce two special devices, trait grouping and top $\gamma_{n,\alpha }$ profile, into the framework, to address the concerns, including the correlation structure among traits and the magnitude of LOD scores, in hotspot detection. In trait grouping, by well using the QTL mapping results, the traits with QTL being localized in the same bins are grouped into the same trait groups, and these QTL are permuted together separately by trait group to cope with correlation structure among traits and obtain stricter thresholds. The top $\gamma_{n,\alpha }$ profile is designed to outline the pattern of top $\gamma_{n,\alpha }$ thresholds for a hotspot across the different EQF architectures constructed by using different LOD score thresholds. If the top $\gamma_{n,\alpha }$ profile of a QTL hotspot shows an increasing (decreasing) pattern, it tells that the hotspot contains relatively fewer (more) QTL with stronger LOD scores, as compared to other hotspots. A flat pattern of top $\gamma_{n,\alpha }$ profile implies that the hotspot contains QTL with balanced LOD scores. Hence, the top $\gamma_{n,\alpha }$ profile can display the relative significance status of a hotspot at different EQF architectures and can characterize and identify the types of QTL hotspots with different hotspot sizes and LOD-score distributions. In this way, our statistical framework can account for the correlation structure among traits and identify the different types of hotspots with very low computational cost in the hotspot detection. Simulation study, numerical analysis, and real examples are used to illustrate the proposed statistical framework, verify the related properties, and compare with the existing methods in the QTL hotspot detection.

It has been pointed out that the spurious hotspots may arise from non-genetic correlations among traits or the use of liberal thresholds in the process of QTL hotspot detection (Darvasi 2003; Perez-Enciso 2004; Neto *et al.* 2012). The non-genetic correlations among traits are capable of inducing a spurious linkage, leading to excessive correlated traits being mapped to the similar regions and creating spurious QTL hotspots. The liberal thresholds arise out of ignoring the correlation structure among traits in the computation of the thresholds when assessing the significance of QTL hotspots (Breitling *et al.* 2008). Both imply the need to take genetic correlations among traits as well as the linkages between the underlying QTL into account for avoiding spurious hotspots in QTL hotspot detection. Linkages have been well known to be the main cause of genetic correlations among traits (Falconer and Mackay 1996). Such a fact is obvious for monogenic traits. However, for the multigenic traits, we show by equations (2–4) that they are not equivalent in the sense that strong linkages will not be necessary to create a significant genetic correlation between traits, simply because different linkage components may individually contribute positive or negative to the genetic correlation, but collectively combine to produce a low or no genetic correlation. This validates the approach to directly considering the linkages between QTL instead of genetic correlations for trait grouping in the analysis to dismiss spurious hotspots. The QTL mapping technique has proven to be powerful in estimating the QTL positions and related parameters to make inference about the linkages among QTL and to dissect the phenotypic correlation into genetic and non-genetic correlations for the traits (Jiang and Zeng 1995; Kao *et al.* 1999). By taking advantage of the QTL mapping results, our framework groups the traits with QTL being localized in the same bins together and permutes these QTL together to dismiss spurious linkages among traits and compute much stricter thresholds, so as to have the ability to control the genome-wide error rates and avoid spurious hotspots. Instead of performing an infeasible multiple-trait joint analysis (Jiang and Zeng 1995) for testing the pleiotropy *vs.* close linkage among numerous QTL and traits, we directly treat the QTL being localized in the same bins as the tightly linked or pleiotropic QTL for trait grouping. In practice, pleiotropic traits cannot be totally grouped together and the traits in different groups may remain correlated to each other (due to linkage). Our method may still suffer from the same problem (inflated error rates and underestimated thresholds) as the Q-method. But, the problem will be subtler because trait grouping can still control the major correlation components to cope with the correlation structure among traits effectively to some extent and result in more conservative thresholds and less spurious hotspots in the detection analysis. Our statistical framework relied on the QTL detected by using appropriate QTL mapping methods. Here, the single-QTL regression interval mapping (Haley and Knott 1992) is adopted to estimate the QTL parameter. It would also be possible to extend our approach to using the EM interval mapping (Lander and Botstein 1989) and multiple-QTL interval mapping methods (Kao *et al.* 1999; Sen and Churchill 2001). With multiple-QTL interval mapping, *e.g.*, using the multiple-QTL mapping functions in QTL Cartographer (Basten *et al.* 1999) or R/qtl (Broman *et al.* 2003), the LOD profile for each QTL adjusted for all other QTL can be obtained and used to construct the QTL matrices given some specified LOD thresholds. Once the QTL matrices are obtained, the subsequent steps are then straightforward to implement in the detection analysis.

The genetical genomics experiment is usually performed to produce the transcript abundance of many genes (the transcription profile) at a time point or under a specific condition in the life stage of an organism. Then, by performing the QTL mapping analysis on the transcription profile followed by the analysis of QTL hotspot detection, we can obtain the EQF architecture (given an LOD score threshold) to outline the QTL hotspot architecture along the genome for the organism. The QTL hotspot architecture actually summarizes the expressivity of genes at all the genomic positions and can be used to infer the networks among QTL hotspots, genes, and traits at a time point for the organism (Yang *et al.* 2019). As the microarray technology for gene expressions becomes less expensive, it is possible to conduct the genetical genomics experiments at several time points during the life cycle of an organism to collect multiple transcription profiles for further investigating the behaviors of the QTL hotspots over the time course of the experiments. To take rice as an example, the genetical genomics experiments can be conducted at the vegetative, reproductive, and ripening stages, or at multiple time points under the abiotic and biotic stresses (such as disease infection, pathogen attack, cold, drought, and salt stresses) to obtain multiple transcription profiles. Then, we can perform the QTL mapping and hotspot detection analysis on the transcription profiles to obtain their respective QTL hotspot architectures at all the time points. Our statistical framework for QTL hotspot detection has a very low computational cost and hence is particular suitable for obtaining the QTL hotspot architectures for all the transcription profiles within a reasonable time frame as compared to the methods by permuting the individual-level data (without bothering the use of parallel computation on a cluster, see Neto *et al.* 2012). The collective QTL hotspot architectures can be used to discern the strengths (the pattern) of each specific QTL hotspot at different life stages or different time points after suffering the stresses. Through investigating the patterns of QTL hotspots across different time points, we can understand how the genes (genomic positions) express themselves differently at the different time points (stages) to outline their dynamic behaviors during the experiments. The study of together using the QTL public databases and the collective QTL hotspot architectures obtained from a series of genetic genomics experiments can help to explore the networks among the expressivity of genes, QTL hotspots, and quantitative traits, as well as to provide deeper insight into the dynamic genomic activity for the organisms in broad areas of biological studies.
